# Distinct roles of short and long thymic stromal lymphopoietin isoforms in house dust mite-induced asthmatic airway epithelial barrier disruption

**DOI:** 10.1038/srep39559

**Published:** 2016-12-20

**Authors:** Hangming Dong, Yahui Hu, Laiyu Liu, Mengchen Zou, Chaowen Huang, Lishan Luo, Changhui Yu, Xuan Wan, Haijin Zhao, JiaLong Chen, Zhefan Xie, Yanqing Le, Fei Zou, Shaoxi Cai

**Affiliations:** 1 Chronic Airways Diseases Laboratory, Department of Respiratory and Critical Care Medicine, Nanfang Hospital, Southern Medical University, Guangzhou, 510515, China; 2 School of Public Health and Tropical Medicine, Southern Medical University, Guangzhou, 510515, China

## Abstract

Loss of airway epithelial integrity contributes significantly to asthma pathogenesis. Thymic stromal lymphopoietin (TSLP) may have dual immunoregulatory roles. In inflammatory disorders of the bowel, the long isoform of TSLP (lfTSLP) promotes inflammation while the short isoform (sfTSLP) inhibits inflammation. We hypothesize that lfTSLP contributes to house dust mite (HDM)-induced airway epithelial barrier dysfunction and that synthetic sfTSLP can prevent these effects. *In vitro*, airway epithelial barrier function was assessed by monitoring transepithelial electrical resistance, fluorescent-dextran permeability, and distribution of E-cadherin and β-catenin. *In vivo*, BALB/c mice were exposed to HDM by nasal inhalation for 5 consecutive days per week to establish an asthma model. sfTSLP and 1α,25-Dihydroxyvitamin D3 (1,25D3) were administered 1 h before HDM exposure. After 8 weeks, animal lung function tests and pathological staining were performed to evaluate asthma progression. We found that HDM and lfTSLP impaired barrier function. Treatment with sfTSLP and 1,25D3 prevented HDM-induced airway epithelial barrier disruption. Moreover, sfTSLP and 1,25D3 treatment ameliorated HDM-induced asthma in mice. Our data emphasize the importance of the different expression patterns and biological properties of sfTSLP and lfTSLP. Moreover, our results indicate that sfTSLP and 1,25D3 may serve as novel therapeutic agents for individualized treatment of asthma.

Asthma is a chronic inflammatory disease of the conducting airways in which many cells of the innate and adaptive immune systems act along with epithelial cells to cause airway inflammation, mucus overproduction, bronchial hyper-reactivity, and airway narrowing^1^,^2^. Airway epithelium has typically been thought to function mainly as the first defensive barrier by impeding the access of allergens^3^. The integrity of the epithelial barrier depends on apical tight junctions composed of zonula occludens 1-3, occludin, and claudin 1-5, and on adherens junctions, which consist of E-cadherin, β-catenin, and junctional adhesion molecule, that keep bronchial epithelial cells together and maintain their apicobasal polarity^4^. E-cadherin is regarded as the ‘gatekeeper’ in the airway mucosa and in allergic sensitization due to its key role in suppressing the production of allergenic mediators and promoting the establishment of tolerance^5^. Structural and functional abnormalities of E-cadherin may lead to enhanced signaling between the epithelium and the underlying immune and structural cells.

Thymic stromal lymphopoietin (TSLP) is a novel interleukin (IL)-7–like cytokine, initially isolated from a murine thymic stromal cell line Z210R.1 and described to be a lymphocyte growth factor^6^. During allergic inflammation, TSLP is produced by various cell types, including epithelial cells, epidermal keratinocytes, dendritic cells (DCs), and mast cells^7^. TSLP may promote immune responses that can be protective or detrimental to the host. On the one hand, TSLP has been connected with protective functions. For example, DCs produce TSLP that acts directly on T-cells by controlling Th17 cell differentiation, fosters regulatory T-cell development, and protects against colitis^8^. Nasal epithelial-derived TSLP can also preserve the mucosal barrier via the upregulation of tight-junction proteins^9^. The C-terminal antimicrobial region of TSLP exerts potent antimicrobial effects^10^. Furthermore, TSLP can regulate the capacity of tolerogenic DCs to drive the differentiation of natural regulatory T cells both in the intestine and thymus^11^,^12^. In contrast to these protective effects, TSLP overexpression can be detected in airway epithelia of asthmatic patients and in mice with asthma^13^,^14^,^15^ and is correlated with the severity of asthma^16^. In addition, TSLP-activated DCs can create a Th2-permissive microenvironment and induce inflammatory Th2 cells that produce the classical Th2 cytokines IL-4, IL-5, and IL-13, and large amounts of tumor necrosis factor (TNF)-α^17^,^18^.

TSLP is present in 2 distinct isoforms, short- and long-form TSLP (hereafter called sfTSLP and lfTSLP, respectively). LfTSLP has 159 amino acids and sfTSLP encompasses the last 63 residues of lfTSLP and is identical to its C-terminal portion^19^. In normal human bronchial epithelial cells, two TSLP splice variants have been reported and lfTSLP, but not sfTSLP, was highly induced after exposure to polyinosinic-polycytidylic acid (polyI:C)^20^. Moreover, in primary human keratinocytes, toll-like receptor ligands, a pro-inflammatory cytokine (TNF-α), and Th2 cytokines (IL-4 and IL-13) predominantly upregulate gene expression of lfTSLP, but not sfTSLP^21^. Another study found that synthetic sfTSLP exerts potent antimicrobial activity and that sfTSLP is the predominant form of TSLP, being the form widely and constitutively expressed in keratinocytes and saliva at steady state, both at the mRNA and protein levels^22^. In inflammatory disorders of the bowel, lfTSLP was shown to promote inflammation and is only expressed during inflammation, while sfTSLP is expressed under steady-state conditions and inhibits inflammation^19^.

In this study, we evaluated the expression of the two TSLP isoforms. Our data showed that lfTSLP is upregulated at the mRNA and protein levels, while sfTSLP is only slightly upregulated, in response to HDM in 16HBE cells. We also found that only lfTSLP contributes to HDM-induced airway epithelial barrier dysfunction and that synthetic sfTSLP prevents this HDM-induced disruption.

## Results

### Effects of HDM on TSLP expression and the barrier integrity of 16HBE cells

**Figure 1:**
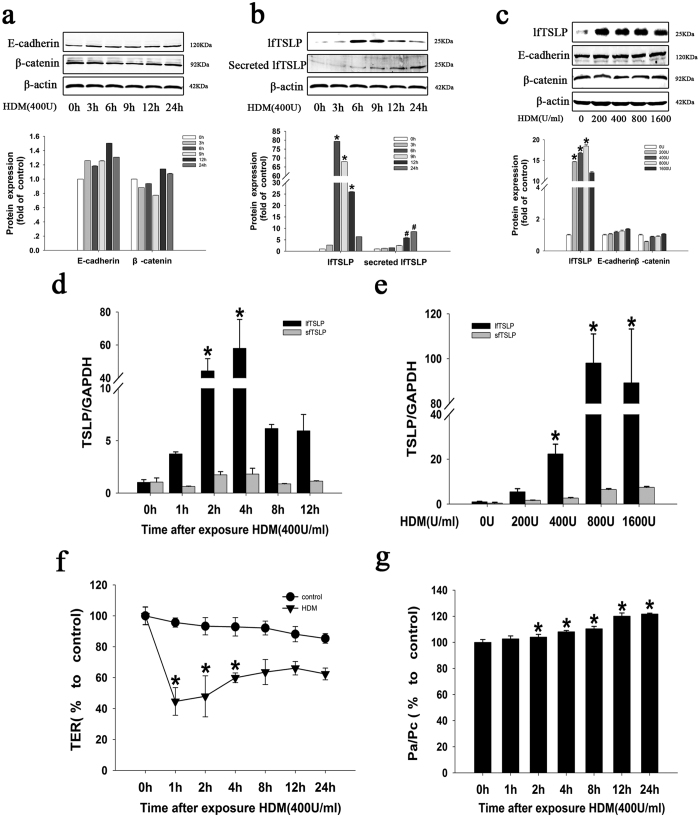
Effects of HDM on the TSLP isoforms expression and the barrier integrity of 16HBE cells. (**a**) The expression of E-cadherin and β-catenin was assessed using western blotting in 16HBE cells stimulated with HDM (400 U) for the indicated times. (**b**) Western blotting was used to detect lfTSLP protein in the cells and the culture medium of 16HBE cells stimulated with HDM for the indicated times. (**c**) Western blotting analysis of E-cadherin, β-catenin, and lfTSLP proteins in 16HBE cells stimulated with different concentrations of HDM for 6 h. (**d**) RT-PCR assay of the long and short splice forms of TSLP mRNA in 16HBE cells stimulated with HDM (400 U) for the indicated times. (**e**) RT-PCR assay of the long and short splice forms of TSLP mRNA in 16HBE cells stimulated with the indicated doses of HDM for 4 h. (**f**) Transepithelial electrical resistance (TER) was measured to determine epithelial barrier integrity after the cells were treated with HDM (400 U) for the indicated times. (**g**) The FITC-Dx permeability was measured to determine epithelial barrier integrity after the cells were treated with HDM (400 U) for the indicated times. Data are presented as the mean ± SD; n = 3–4.*P < 0.05 vs. the control group.

A previous study demonstrated that the expression of lfTSLP, which is a crucial cytokine for the induction of inflammatory Th2 responses, is highly induced by dsRNA in bronchial epithelial cells^20^. Initially, we found a significant upregulation of lfTSLP at the protein (Fig. 1b,c) and mRNA (Fig. 1d,e) levels. Unexpectedly, the mRNA expression of sfTSLP was scarcely induced (Fig. 1d,e) after stimulation with HDM in 16HBE cells. Next, we examined the effects of HDM on barrier permeability in monolayers of 16HBE cells. In the treated 16HBE cells, HDM exposure resulted in TER reduction (Fig. 1f), while the permeability increased (Fig. 1g) in a time-dependent manner. The expression of E-cadherin and β-catenin proteins was not altered (Fig. 1a,c).

### 1,25D3 upregulates sfTSLP expression and inhibits HDM-induced barrier disruption in 16HBE cells

**Figure 2:**
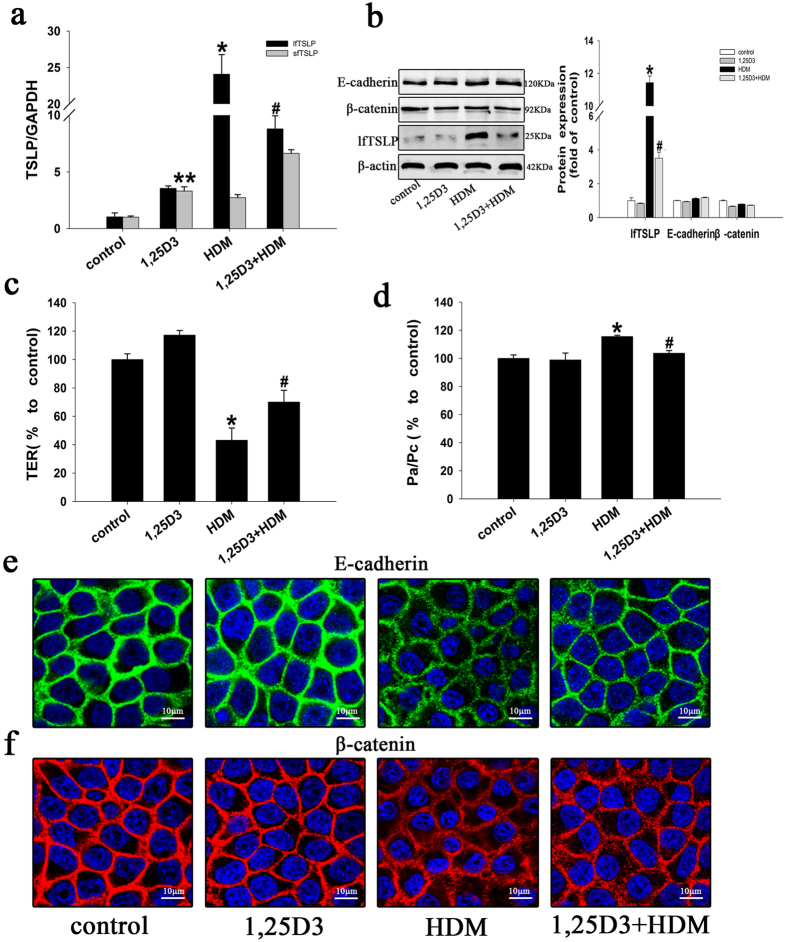
Effect 1,25D3 on the HDM-induced TSLP isoforms expression and the HDM-induced barrier disruption. 16HBE cells were treated with culture medium (control group), 1,25D3 (10 nM), or HDM (400 U) with or without 1,25D3 for 1 h prior to stimulation with HDM. (**a**) RT-PCR assay of the long and short splice forms of TSLP mRNA in the treated cells. (**b**) The expression of E-cadherin, β-catenin, and lfTSLP proteins was assessed using western blotting. (**c**) TER and (**d**) FITC-Dx permeability was measured in the treated cells. (**e**) The distribution of E-cadherin was monitored using immunofluorescence. Green represents E-cadherin, blue represents the nucleus. (**f**) The distribution of β-catenin was monitored using immunofluorescence. Red represents β-catenin, blue represents the nucleus. Data are presented as the mean ± SD; n = 3–4. *P < 0.05 the HDM group vs. the control group; ^#^P < 0.05 the 1,25D3+HDM group vs. the HDM group; **P < 0.05 the 1,25D3 group vs. the control group.

Recent studies show that 1,25D3 plays a positive role in the airway epithelia barrier function^23^,^24^. Therefore, we assessed the effects of 1,25D3 in HDM-treated 16HBE cells. The 16HBE cells were treated with culture medium (control group), 1,25D3 (10 nM)^24^, or HDM (400 U), with or without 1,25D3 for 1 h. We found that 1,25D3 upregulated sfTSLP expression and inhibited HDM-dependent upregulation of lfTSLP (Fig. 2a,b) and that 1,25D3 treatment significantly reversed the effects of HDM, including the decrease in TER (Fig. 2c), increase in fluorescein isothiocyanate (FITC) - dextran (Dx) permeability (Fig. 2d), and the delocalization of E-cadherin (Fig. 2e) and β-catenin (Fig. 2f).

### Mediation of HDM-induced lfTSLP upregulation by the mitogen-activated protein kinase (MAPK) signaling cascade

**Figure 3:**
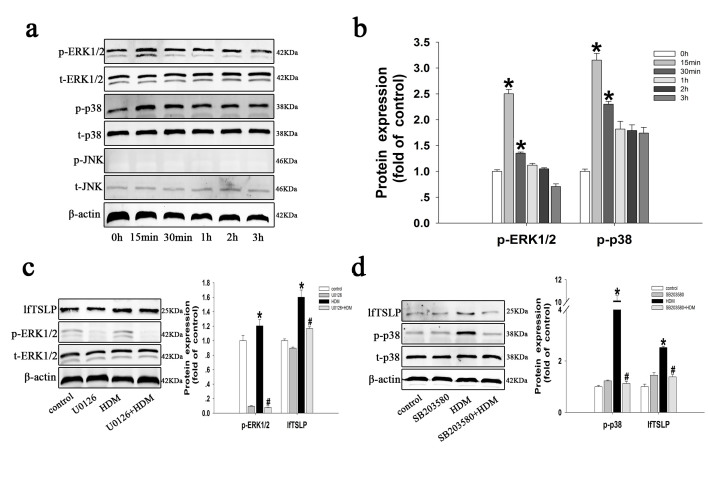
HDM-induced lfTSLP upregulation is mediated by the MAPK signaling cascade in 16HBE cells. (**a,b**) Activation of three MAPK pathway proteins, (phosphorylated [p]-MAPK/total [t]-MAPK], p-ERK:t-ERK, p-p38:t-p38, and p-c-Jun N-terminal kinase (JNK):t-JNK was measured by western blotting and densitometric analyses in 16HBE cells stimulated with HDM (400 U) for the indicated times. (**c**) 16HBE cells were respectively treated with culture medium (control group), U0126 (10 μM), or HDM (400U) with or without U0126 (10 μM) for 1 h prior to stimulation with HDM. The expression of p-ERK, t-ERK, and lfTSLP proteins was evaluated using western blotting analysis in the treated cells. (**d**) 16HBE cells were respectively treated with culture medium (control group), SB203580 (10 μM), or HDM (400 U) with or without SB203580 (10 μM) for 1 h prior to stimulation with HDM. The expression of p-p38, t-P38, and lfTSLP protein was evaluated using western blotting analysis in the treated cells. Data are presented as the mean ± SD; n = 3–4. *P < 0.05 vs. the control group; ^#^P < 0.05 vs. the HDM group.

MAPKs are involved in diverse cell-mediated initiation by various stimuli, such as pro-inflammatory cytokines and growth factors^25^. In our study, HDM induced a rapid and marked phosphorylation of ERK1/2 and p38, but not c-Jun N-terminal kinase (JNK). Western blotting analysis showed that phosphorylation of ERK1/2 and p38 reached a maximum at 15–30 min after HDM exposure (Fig. 3a,b). These data indicate that HDM activates the ERK1/2 and p38 signaling pathways in 16HBE cells. To investigate the role of MAPK signaling pathway components in HDM-induced lfTSLP expression, we used SB203580 and U0126, specific and potent inhibitors of p38 and p42/p44 ERK, respectively. Treatment of 16HBE cells with SB203580 or U0126 1 h before stimulation with HDM led to a significant inhibition of lfTSLP expression (Fig. 3c,d). Taken together, these results indicate that activation of the MAPK pathway (ERK1/2 and p38) is essential for HDM-mediated lfTSLP expression in16HBE cells.

### Involvement of STAT5 phosphorylationin lfTSLP-induced barrier dysfunction

**Figure 4:**
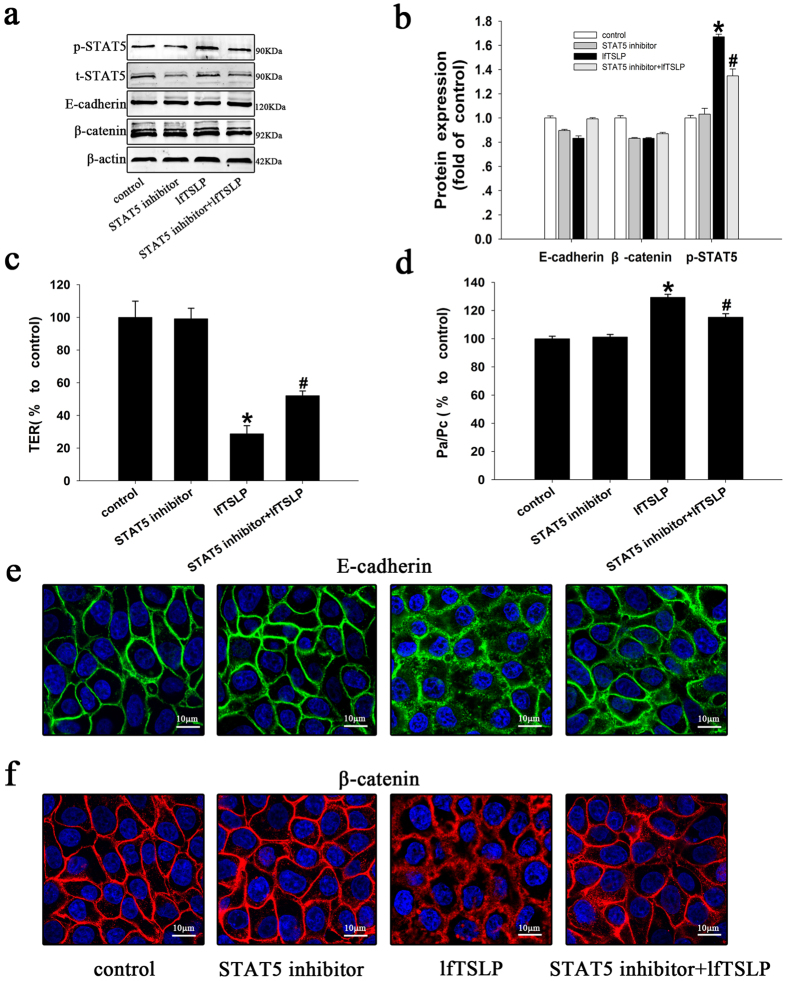
Involvement of STAT5 phosphorylationin lfTSLP-induced barrier dysfunction in 16HBE cells. 16HBE cells were treated with culture medium (control group), STAT5 inhibitor (10 μM), or lfTSLP (10 ng/ml) with or without STAT5 inhibitor (10 μM) for 1 h prior to stimulation with lfTSLP. (**a,b**) p-STAT5, t-STAT5, E-cadherin, and β-catenin protein expression was evaluated by western blotting and densitometric analyses. (**c**) TER and (**d**) FITC-Dx permeability was measured in the treated cells. (**e**) The distribution of E-cadherin was monitored using immunofluorescence. Green represents E-cadherin, blue represents the nucleus. (**f**) The distribution of β-catenin was monitored using immunofluorescence. Red represents β-catenin, blue represents the nucleus. Data are presented as the mean ± SD; n = 3–4. *P < 0.05 vs. the control group; ^#^P < 0.05 vs. the lfTSLP group.

Previous studies have demonstrated that TSLP binds with TSLP receptor (TSLPR) to induce epithelial cell proliferation and wound healing through the phosphorylation of signal transducer and activator of transcription (STAT)5 in bronchial epithelial cells^26^. Firstly, our results show that treatment with lfTSLP (10 ng/ml) induces more marked abnormalities in TER (Fig. 4c), FITC-Dx permeability (Fig. 4d) and delocalization of E-cadherin (Fig. 4e) and β-catenin (Fig. 4f). As a mechanism responsible for the lfTSLP-induced barrier dysfunction, we proposed that the phosphorylation of STAT5 may contribute to this type of barrier dysfunction. We observed a significant and reproducible increase in STAT5 tyrosine phosphorylation when the 16HBE cells were stimulated with lfTSLP (Fig. 4a,b). To further establish the role of STAT5 activation, we assessed airway epithelial barrier function in the presence of a STAT5 inhibitor. Compared with the control groups, treatment with the STAT5 inhibitor partial reversed the lfTSLP-induced changes in TER and FITC-Dx permeability (Fig. 4c,d), and facilitated redistribution of E-cadherin (Fig. 4e) and β-catenin (Fig. 4f) and phosphorylation of STAT5 (Fig. 4a,b). These data suggest that STAT5 signaling contributes to the lfTSLP-induced dysfunction of the epithelial barrier.

### Protective effect of sfTSLP on HDM- and lfTSLP-induced airway epithelial barrier disruption

**Figure 5:**
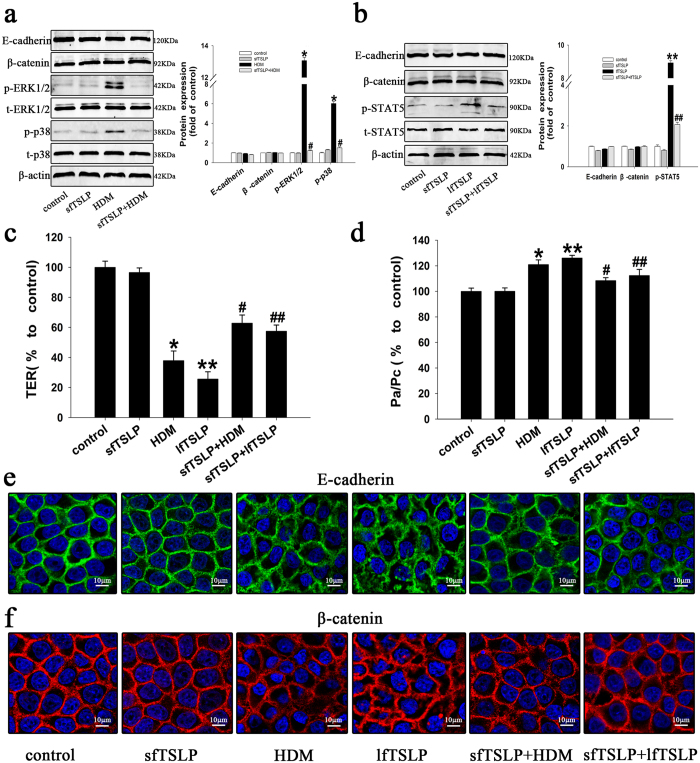
Protective effect of sfTSLP on HDM- and lfTSLP-induced airway epithelial barrier disruption in 16HBE cells. (**a**) 16HBE cells were treated with culture medium (control group), sfTSLP (100 ng/ml), or HDM (400 U) with or without sfTSLP (100 ng/ml) for 1 h prior to stimulation with HDM. The expression of p-ERK, t-ERK, p-p38, t-P38, E-cadherin, and β-catenin proteins was assessed using western blotting analysis. (**b**) 16HBE cells were treated with culture medium (control group), sfTSLP (100 ng/ml), or lfTSLP (10 ng/ml) with or without sfTSLP (100 ng/ml) for 1 h prior to stimulation with lfTSLP. The expression of p-STAT5, t-STAT5, E-cadherin, and β-catenin proteins was assessed using western blotting. (**c**) TER and (**d**) FITC-Dx permeability was measured in the different six groups of treated cells. (**e**) The distribution of E-cadherin was monitored using immunofluorescence. Green represents E-cadherin, blue represents the nucleus. (**f**) The distribution of β-catenin was monitored using immunofluorescence. Red represents β-catenin, blue represents the nucleus. Data are presented as the mean ± SD; n = 3–4.*P < 0.05 the HDM group vs. the control group; ^#^P < 0.05 the sfTSLP+HDM group vs. the HDM group; **P < 0.05 the lfTSLP group vs. the control group; ^##^P < 0.05 the sfTSLP+lfTSLP vs. the lfTSLP group.

A previous study showed that vitamin D3 induced increased transcription primarily of the sfTSLP isoform and that sfTSLP exerts anti-inflammatory effects in inflammatory disorders of the bowel^19^. In our previous results, we have found that 1,25D3 upregulated sfTSLP expression. Therefore, we postulated that sfTSLP might prevent HDM-induced airway epithelial barrier disruption. To test this hypothesis, we chemically synthesized sfTSLP and then treated 16HBE cells with culture medium (control group), sfTSLP (100 ng/ml), or HDM (400 U) with or without sfTSLP for 1 h prior to stimulation with HDM. Treatment with sfTSLP not only reduced the expression of HDM-induced phosphorylation of ERK1/2 and p38 (Fig. 5a), but also decreased the changes in TER (Fig. 5c), FITC-Dx permeability (Fig. 5d), and delocalization of E-cadherin (Fig. 5e) and β-catenin (Fig. 5f). Interestingly, in contrast to the effects of lfTSLP, sfTSLP also partially reversed lfTSLP-induced TER reduction (Fig. 5c), permeability increase (Fig. 5d), and E-cadherin (Fig. 5e) and β-catenin (Fig. 5f) redistribution. sfTSLP did not induce STAT5 phosphorylation (Fig. 5b). However, when 16HBE cells were stimulated with the two isoforms, either alone or together, sfTSLP inhibited lfTSLP-dependent STAT5 phosphorylation (Fig. 5b). These data indicate that sfTSLP plays a protective role on HDM-induced airway epithelial barrier disruption.

### SfTSLP and 1,25D3 decrease airway hyper-reactivity and inflammation in the mouse model of HDM-induced asthma

**Figure 6:**
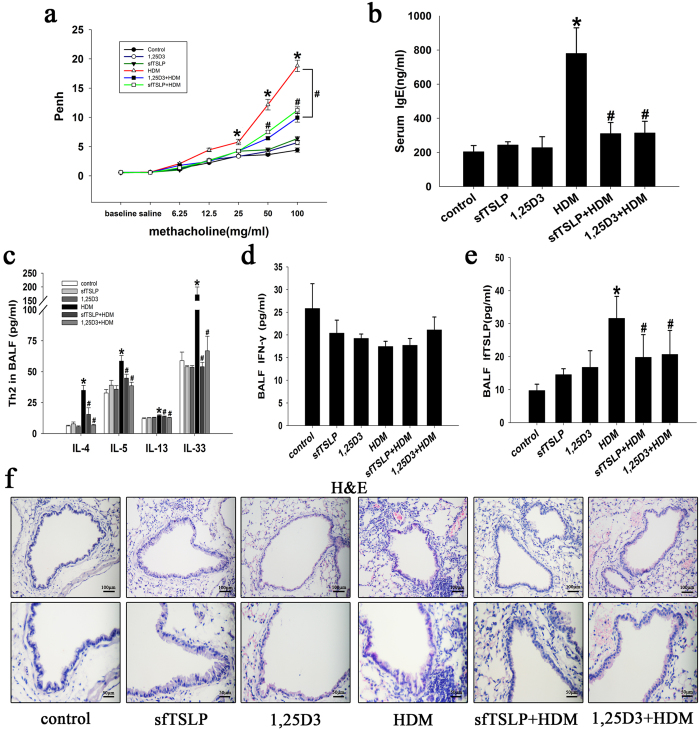
sfTSLP and 1,25D3 attenuate airway hyper-reactivity and airway inflammation in the mouse model of HDM-induced asthma. (**a**) Airway hyper-responsiveness was measured by whole body plethysmography. (**b**) IgE levels in serum were measured by ELISA. (**c**) IL-4, IL-5, IL-13, and IL-33 levels in BALF were measured by ELISA. (**d**) IFN-γ levels in BALF were measured by ELISA. (**e**) lfTSLP levels in BALF were measured by ELISA. (**f**) Representative hematoxylin/eosin-stained lung sections from the different groups. Magnification, 200× (upper panel) and 400× (lower panel). Data are presented as the mean ± SE; n = 5–8/group.*P < 0.05 vs. the control group; ^#^P < 0.05 vs. the HDM group.

To assess the validity of the mouse model of HDM-induced, we monitored the enhanced pause (Penh) response to assess airway reactivity to methacholine. The Penh values were significantly increased in the HDM-treated mice, compared with the saline-treated control group following stimulation with 25, 50, and 100 mg/ml methacholine (Fig. 6a). Treatment with sfTSLP and 1,25D3 significantly inhibited the AHR to levels similar to that in mice that inhaled methacholine (Fig. 6a). Concomitantly, serum IgE levels were markedly raised by HDM, and this effect was inhibited by sfTSLP and 1,25D3 treatment (Fig. 6b). Next, we analyzed the levels of Th1/Th2-associated cytokines in the BALF of mice. The BALF IL-4, IL-5, IL-13, IL-33 (Th2-related; Fig. 6c), and lfTSLP (Fig. 6e) levels, but not IFN-γ (Th1-related; Fig. 6d) was increased in HDM-exposed mice, compared with PBS-treated control animals. These data support the model that asthmatic airway inflammation induced by HDM is a robust Th2 response in mice, and that sfTSLP and 1,25D3 markedly suppress the dominant production of Th2-associated cytokines and the upregulation of lfTSLP in BALF. Histological examination of lung sections from HDM-treated mice indicated markedly large numbers of infiltrating inflammatory cells in the peribronchial regions, as well as evident epithelial hyperplasia and a degree of epithelial shedding, compared to the control group, while treatment with sfTSLP and 1,25D3 mitigated this HDM-induced peribronchial inflammation (Fig. 6f).

### SfTSLP and 1,25D3 ameliorate HDM-induced redistributionof E-cadherin and β-catenin and upregulation of lfTSLP in airway epithelia

**Figure 7:**
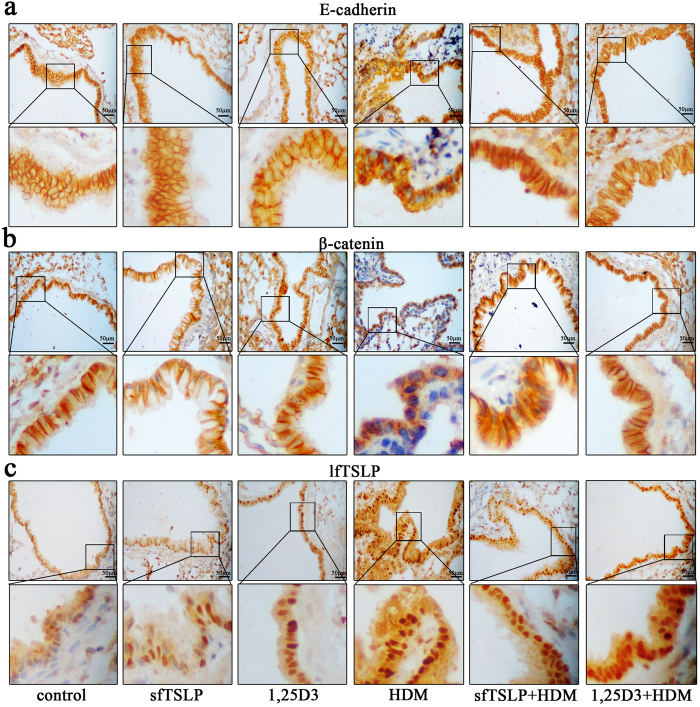
sfTSLP and 1,25D3 ameliorate the HDM-induced redistribution of E-cadherin and β-catenin and the upregulation of lfTSLP in bronchial tissue. (**a**) Representative immunohistochemical staining images of E-cadherin in bronchial tissue. Magnification, 400×. (**b**) Representative immunohistochemical staining images of β-catenin in bronchial tissue. Magnification, 400×. (**c**) Representative immunohistochemical staining images of lfTSLP in bronchial tissue. Magnification, 400×. n = 5–8/group.

The lung sections analyzed using immunofluorescence staining presented a strong immunoreactivity of E-cadherin, β-catenin, and lfTSLP in airway epithelial cells. Exposure to HDM resulted in aberrant distribution of E-cadherin (Fig. 7a) and β-catenin (Fig. 7b) at the epithelial cell-cell contact sites and much stronger staining for lfTSLP (Fig. 7c) in airway epithelial cells. These effects were partially reversed by treatment with sfTSLP and 1,25D3 (Fig. 7a–c).

### SfTSLP and 1,25D3 inhibit the HDM-induced downregulation in E-cadherin and β-catenin and phosphorylation of ERK1/2 and p38

**Figure 8:**
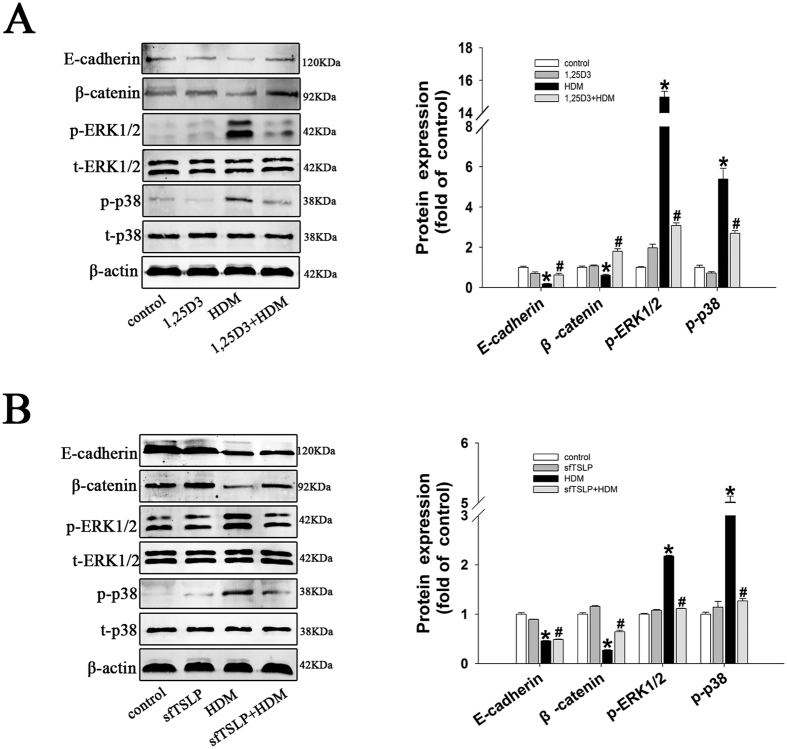
sfTSLP and 1,25D3 inhibit the HDM-induced downregulation in E-cadherin and β-catenin and phosphorylation of ERK1/2 and P38 in lungs. (**A**) Western blotting analysis of p-ERK, t-ERK, p-p38, t-P38, E-cadherin, and β-catenin proteins in whole lung homogenates in the control, 1,25D3, HDM, and 1,25D3+HDM groups. (**B**) Western blotting analysis of p-ERK, t-ERK, p-p38, t-P38, E-cadherin, and β-catenin proteins in whole lung homogenates in the control, sfTSLP, HDM, and sfTSLP+HDM groups. Data are presented as the mean ± SD; n = 5/group. *P < 0.05 vs. the control group; ^#^P < 0.05 vs. the HDM group.

We next investigated whether HDM affects the expression of E-cadherin and β-catenin proteins in lung. Western blotting analysis indicated a significant decrease in total expression of E-cadherin and β-catenin proteins in lungs of HDM-treated mice and partial reversal of this downregulation upon treatment with sfTSLP and 1,25D3 (Fig. 8A,B). Consistent with the HDM-induced changes seen in16HBE cells, there was a marked increase in the expression of phosphorylated (p)-ERK1/2 and p-p38 in the lungs of HDM-treated mice, which was significantly reversed by treatment with sfTSLP and 1,25D3 (Fig. 8A,B).

## Discussion

In this study, we have shown that the two isoforms of TSLP have different expression patterns and biological properties in HDM-induced asthmatic airway epithelial barrier disruption. To our knowledge, ours is the first study to demonstrate that lfTSLP damages the airway epithelial barrier, contributing to the pathogenesis of asthma, and that sfTSLP alleviates HDM-induced airway inflammation and prevents epithelial barrier disruption.

Asthma is one of the most common chronic inflammatory diseases of the airways^2^. Consistent with the findings of previous experimental models^27^,^28^, we successfully established a chronic asthmatic experimental mouse model using repeated, prolonged intranasal administration of HDM. The HDM-exposed mice developed typical asthmatic features, including airway hyper-reactivity, airway inflammation, increased serum IgE levels, as well as an imbalanced Th1/Th2 response.

The primary function of airway epithelium is to serve as a physical barrier for the underlying tissue that responds to the external environment^29^,^30^. The essential features that contribute to airway epithelial barrier dysfunction are loss of the cell-cell contact integrity, disruptions of the coordinate expression and interaction of proteins in cell-cell junctional complexes, especially adherens junctions. Adherens junctions, comprised of E-cadherin, β-catenin, and α-catenin, mechanically connect adjacent cells and initiate the formation and maturation of cell-cell contacts^30^,^31^,^32^. In our study, we selected the airway epithelial cell line 16HBE to assess the effects of HDM and lfTSLP on barrier properties *in vitro*. Compared with the effects of external stimuli on barrier function^23^,^24^,^33^, HDM caused an increase in both ionic and macromolecular permeability (TER reduction and permeability increase) in association with the E-cadherin and β-catenin cleavage. Subsequently, the *in vivo* experiments and in the mouse model showed an HDM-induced aberrant arrangement of E-cadherin and β-catenin at epithelial cell-cell contact sites. However, the expression of E-cadherin and β-catenin proteins in HDM-treated 16HBE cells remained unchanged, while that in lungs of HDM-treated mice was decreased. A reasonable explanation for the discrepancy in the cultured cells vs. the mouse model is that in the mice, the airway inflammation and the damage to epithelial barrier function influence each other. HDM-induced airway inflammation is persistent, which may cause greater damage to epithelial barrier function, causing liberation of larger amounts of adhesive proteins like E-cadherin and β-catenin from the membrane.

TSLP is produced by epithelial cells and has pleiotropic biological functions. Recent studies have addressed the role of the two isoforms of TSLP. The two isoforms of TSLP are expressed in many human tissues; while sfTSLP mRNA is constitutively expressed, lfTSLP mRNA is not^20^. Only lfTSLP was markedly induced by invasive bacteria or pro-inflammatory stimuli in normal human airway epithelial cells, primary human keratinocytes and intestinal epithelial cells^19^,^20^,^21^. The present study shows that 16HBE cells express lfTSLP only in response to HDM, whereas the expression of sfTSLP remains unaltered. We previously reported that 1,25D3 alleviates toluene diisocyanante- and cigarette smoke-induced airway inflammation and prevents epithelial barrier disruption^23^,^24^. In the current study, we also found that 1,25D3 inhibits HDM-induced asthmatic airway epithelial barrier disruption. Moreover, 1,25D3 upregulates sfTSLP expression and inhibits HDM-dependent upregulation of lfTSLP. These data are consistent with earlier studies that showed that the expression of sfTSLP is constitutive and can be upregulated by anti-inflammatory mediators (vitamin D3) in skin keratinocytes^19^ and that TSLP, predominantly sfTSLP, is upregulated by VDR agonists^21^. Hence, upregulation of sfTSLP may be one of the mechanisms of vitamin D-mediated protection against airway inflammation, supporting its advantages in the treatment of asthma^34^,^35^. sfTSLP has been confirmed to exert potent antimicrobial activity^22^ and have anti-inflammatory activities in inflammatory disorders of the bowel^19^. At the protein level, sfTSLP encompasses the last 63 residues of lfTSLP. We synthesized the sfTSLP peptide and found that treatment with sfTSLP prevents HDM-induced asthmatic airway epithelial barrier disruption and alleviates airway inflammation *in vitro* and *in vivo*. Nevertheless, the mechanisms by which sfTSLP enhances barrier function in HDM-induced airway epithelial barrier disruption remain to be elucidated.

MAPKs are well-known cell cycle regulators that convert extracellular stimuli into a wide range of cellular responses, and the ERK, JNK, and p38 MAPK are three well-characterized MAPK pathways^25^. Previous studies showed that IL-33-induced TSLP expression is mediated by MAPK signaling, via ERK, JNK, and p38 activation^36^ and that allergen-induced IL-25 and TSLP production were regulated via MAPK pathways^37^. In this study, HDM significantly induced the expression of p-ERK and p-p38, but not p-JNK. To further elucidate the relationship between the HDM-dependent upregulation of lfTSLP and the MAPK signaling pathway, we treated 16HBE cells with HDM in the presence of ERK and p38 inhibitors and found that both inhibitors reversed the HDM-mediated increase in lfTSLP. These data suggest that HDM induces lfTSLP expression via the ERK and p38 signaling pathways.

We also found that lfTSLP causes more severe damage to epithelial barrier function than HDM, suggesting that lfTSLP plays key roles in HDM-induced asthma. The mechanisms through which lfTSLP disrupt the airway epithelial barrier warrant further studies. Recent studies have demonstrated that TSLP promotes transcription of target genes via STAT5 activation^26^,^38^. Bronchial epithelial cells express TSLPR, which interacts with TSLP to induce cell proliferation and injury repair mediated by STAT5 phosphorylation^26^. In the present study, we showed that STAT5 inhibition prohibits lfTSLP-induced airway epithelial barrier disruption, indicating that lfTSLP-induced damage in asthma is STAT5-dependent. These data support the model that HDM exposure upregulates lfTSLP through the ERK and p38 signaling pathways and subsequently, lfTSLP contributes to airway epithelial barrier dysfunction via STAT5 phosphorylation.

Intriguingly, we found that sfTSLP can attenuate HDM- and lfTSLP-induced airway epithelial barrier disruption. In addition, sfTSLP significantly inhibited the activation of the ERK and p38 signaling pathways by HDM and the lfTSLP-induced phosphorylation of STAT5. However, a previous study that showed that sfTSLP does not block or inhibit lfTSLP-dependent STAT5 phosphorylation^19^. The different cell types utilized in these studies might explain the contradictory results. Our results demonstrate that sfTSLP prevents HDM-induced airway epithelial barrier disruption, and that the inhibition of the ERK/p38 pathway and the lfTSLP/TSLPR/STAT5 pathway may be involved in this process. However, the role of the interaction between sfTSLP and lfTSLP and the heterodimeric receptor IL-7Rα/TSLPR complex in this process needs to be investigated in future studies.

In conclusion, we demonstrated 2 distinct roles for the long and short TSLP isoforms; lfTSLP contributes to HDM-induced airway epithelial dysfunction, whereas synthetic sfTSLP exerts protective effects in HDM-induced airway inflammation and in the asthmatic mouse model. Moreover, our findings suggest that sfTSLP is a candidate for further development as a personalized therapeutic agent for the prevention and treatment of patients with asthma. Finally, our data emphasize the need for analyzing the roles of the two TSLP isoforms separately in future studies.

## Methods

### Preparation of TSLP peptides

Synthetic sfTSLP peptides (63aa: MFAMKTKAALAIWCPGYSETQINATQAMKKRRKRKVTTNKCLEQVSQLQGLWRRFNRPLLKQQ) were prepared by China Peptides (Shanghai, China). Recombinant Human TSLP (lfTSLP) was obtained from R&D systems.

### Animals and experimental protocol

Specific-pathogen-free BALB/c mice (male, 6–8 weeks old, 20–24 g) were purchased from Southern Medical University (Guangzhou, China). The mice were housed in a specific pathogen-free environment (room temperature 24 °C, humidity range 40–70%, and a 12-h light/dark cycle). Sterilized water and food were provided ad libitum. The mice used in our study were treated according to an experimental plan approved by the committee of Southern Medical University on the use and care of animals. All procedures complied with the guidelines of the Institutional Animal Ethics Committee for the care and use of laboratory animals. Purified HDM extract was purchased from ALK-Abello A/S (Denmark) and 1α,25-dihydroxyvitamin D3 (1,25D3; molecular weight, 416.64) was obtained from Sigma (USA). Mice were randomly placed into one of the following six groups: (1) control group, in which the mice received phosphate-buffered saline (PBS; Gibco, Life Technology); (2) sfTSLP group; (3) 1,25D3 group; (4) HDM group; (5) sfTSLP+HDM group, in which mice were pretreated with sfTSLP, followed by HDM; and (6) 1,25D3+HDM group, in which the mice were pretreated with 1,25D3, followed by HDM. All treatments (except 1,25D3) were administered via intranasal inhalation. 1,25D3 was administered to the mice by intraperitoneal injection.

Briefly, mice were exposed to intranasal sevoflurane-anesthesia, then treated with 10 μL PBS, HDM (400 U/mouse each day, dissolved in de-ionized water), sfTSLP (0.1 μg/μL, dissolved in deionized water) and 300 μL 1,25D3 (100 ng/mouse, dissolved in 300 μL PBS containing 0.9% ethanol). In the sfTSLP+HDM and 1,25D3+HDM groups, the anaesthetized mice were pretreated with sfTSLP and 1,25D3 60 min prior to the administration of HDM. These treatment procedures were carried out daily for 5 consecutive days, followed by two days of rest, for 8 consecutive weeks.

### Assessment of airway hyper-responsiveness (AHR) to methacholine

Airway parameters were measured 24 h after the last challenge. Mice were placed in a barometric plethysmographic chamber (Buxco Electronics, Troy, NY). The mice were first placed in a chamber for acclimatization, the baseline response was determined, and then normal saline was nebulized into the mice, followed by the administration of increasing concentrations (6.25, 12.5, 25, 50, and 100 mg/ml, respectively) of nebulized methacholine (Sigma Aldrich) in tandem. The reading interval was set to 5 min following each nebulization. The bronchopulmonary resistance was expressed as enhanced pause (Penh).

### Enzyme-linked immunosorbent assay (ELISA) for serum IgE and analysis of bronchoalveolar lavage fluid (BALF)

As previously described^39^, mice were sacrificed with pentobarbital (100 mg/kg, *i.p.*) one day after the last airway challenge. Blood samples were allowed to rest for 2 h at room temperature, then centrifuged (3000 × *g*, 20 min), and supernatants were harvested and stored at −80 °C. Total serum IgE was measured by ELISA (eBioscience, San Diego, US) according to the manufacturer’s instructions. Next, the lungs were lavaged twice *in situ* with 0.8 ml sterile saline (0.9% NaCl, pre-warmed), and the recovered fluid was pooled. Then, the fluids were centrifuged (1000 × *g*, 10 min) and supernatants were stored for further analysis of IFN-γ, IL-4, IL-5, IL-13, IL-33, and TSLP(lfTSLP) by ELISA (eBioscience) according to the manufacturer’s instructions.

### Pulmonary histologic examination

Left lungs were gently infused with 4% neutral formalin to fully inflate all lobes (inflation was judged visually) and immersed in formalin for at least 24 h, then fixed, paraffin-embedded, cut in 4-μm sections, and stained with hematoxylin and eosin (H&E) for blinded histopathologic assessment. For immunohistochemistry of lfTSLP, E-cadherin and β-catenin, lung sections (4 μm) were prepared with a Leica microtome 2030 (Leica Microsystems Nussloch GmbH, Nussloch, Germany), and then submerged in citrate buffer (pH 6.0) for antigen retrieval. Samples were treated with H_2_O_2_ for 15 min to block endogenous peroxidases, and then incubated overnight at 4 °C in recommended dilutions of anti-TSLP (Abcam), anti-E-cadherin (Santa Cruz), and anti-β-catenin (Santa Cruz) antibodies. After washing with PBS, slices were incubated with a secondary antibody for 30 min at room temperature. Signals were visualized with a DAB peroxidase substrate kit (ZhongShanJinQiao, Beijing).

### Cell culture and treatment

The human bronchial epithelial cell line, 16HBEo- (16HBE; Shanghai Fuxiang Biological Technology Co. Ltd., ATCC, USA) was grown in RPMI 1640 medium (Gibco) with 10% fetal bovine serum (Gibco) and placed in a humidified incubator at 37 °C with an atmosphere of 5% CO_2_. When the cells reached 80–90% confluence, the cells were treated with trypsin and seeded into culture plates at a density of 10^4^–10^5^ cells per cm^2^ for use in the experiments. The medium was changed to serum-free RPMI 1640 when the cells reached 85% confluence, and after 12 h, the cells were rinsed with PBS and then stimulated with HDM for the indicated times and doses. The cells were also treated with other mediators and inhibitors, namely lfTSLP, the extracellular signal-regulated kinase (ERK)1/2 tyrosine kinase inhibitor U0126 (10 μM; Cell Signaling Technology, USA), the p38 kinase inhibitor SB203580 (10 μM; Cell Signaling Technology), the STAT5 Inhibitor (10 μM; Santa Cruz, USA), sfTSLP, and 1,25D3 for 1 h prior to stimulation with HDM and lfTSLP in serum-free medium.

### Evaluation of epithelial barrier function

Epithelial barrier function was assessed by measuring TER and FITC-Dx flux across the monolayers of cultured epithelial cells. TER and FITC-Dx flux was quantified as previously described^23^. Briefly, confluent monolayers of 16HBE cells, polarized at an air-liquid interface, were cultured in 12-well Transwell inserts (Corning Costar). TER was measured using a Millicell ERS-2 Epithelial Volt-Ohm meter with an STX01 electrode (Millipore Corp, Billerica, MA, USA). Then, the apical medium (luminal side) was replaced with 200 μL of phenol red-free RPMI 1640 containing 0.5 mg/ml FITC-Dx (Sigma Chemical Co, USA), and the basal medium (non-luminal side) was replaced with 800 μL of phenol red-free RPMI 1640 without FITC-Dx, and the cells were incubated at 37 °C for 90 min. Samples were analyzed by fluorimetry (excitation 492 nm; emission 530 nm). Epithelial permeability was expressed as the percent leakage of FITC-Dx from apical to basolateral compartments.

### Immunofluorescence

At the end of the treatment period, the cells were fixed with 4% paraformaldehyde at room temperature for 10 min, washed with PBS for 30 min, incubated with 0.2% Triton X-100 in PBS for 10 min, and rinsed again with PBS. Cells were blocked with 3–5% BSA in PBS for 2 h. The cell monolayers were then incubated overnight with a primary antibody at 4 °C in PBS containing 3–5% BSA, primary rabbit anti-E-cadherin (Santa Cruz) and anti-β-catenin (Santa Cruz) antibodies. Following PBS washes, the cell monolayers were incubated with a secondary antibody, Alexa Fluor 488 (R37118) or Alexa Fluor 594 (R37119) (1:200 diluted in PBS) (Invitrogen, USA), for 1 h at room temperature in the dark. The cell nuclei were stained with 4′,6-diamidino-2-phenylindole dihydrochloride (Sigma Aldrich) for 10 min. The labeled sections were viewed and images captured using a confocal microscope (FV1000, Olympus) at 100× objective magnification.

### Western blotting analysis

The treated cells and/or the completely homogenized right lung tissue samples were centrifuged and resuspended in lysis solution containing lysis buffer, the protease inhibitor phenylmethylsulfonyl fluoride, and calcineurin inhibitors for 30 min at 4 °C. The samples were centrifuged at 13000 rpm for 15 min, and the supernatants were collected and boiled with standard SDS sample buffer. The samples were resolved by SDS-PAGE and western blotting analysis was performed for detection of the following antigens: secreted TSLP (lfTSLP) (Abcam) in CM(condition medium). TSLP (lfTSLP) (Abcam), p-ERK1/2 (Cell signaling technology), ERK1/2 (Cell signaling technology), p-p38 (Cell signaling technology), p38 (Cell signaling technology), p-JNK (Cell signaling technology.), JNK (Cell signaling technology), p-STAT5 (Cell signaling technology), STAT5 (Cell signaling technology), E-cadherin (Santa Cruz), and β-catenin (Santa Cruz). After incubation with a secondary antibody, signal intensities were analyzed by using the Odyssey infrared Image System (LiCor, USA). The densitometry results were normalized with that of β-actin.

### Quantitative real-time PCR (RT-PCR)

Total RNA was extracted from the treated cells using Trizol (Takara, Japan). RNA samples were then reverse transcribed into first-strand cDNA using the PrimeScriptTM RT reagent kit (Takara). Reverse transcription polymerase chain reaction (RT-PCR) was done following the manufacturer’s instructions by Mx3005 P RealTime PCR Detection System (Stratagene, Santa Clara, CA, USA). Primers for amplifying glyceraldehyde 3-phosphate dehydrogenase (GAPDH) cDNA were 5′-ATCAGCAATGCCTCCTGCAC-3′ (forward) and 5′-TGGCATGGACTGTGGTCATG-3′ (reverse); primers for lfTSLP cDNA were 5′-CACCGTCTCTTGTAGCAATCG-3′ (forward) and 5′-TAGCCTGGGCACCAGATAGC-3′ (reverse); primers for sfTSLP cDNA were 5′-cgtaaactttgccgcctatga-3′ (forward) and 5′-ttcttcattgcctgagtagcatttat-3′ (reverse). The amplification protocol was set as follows: denaturation at 95 °C for 10 min; 40 or 50 cycles denaturation at 95 °C for 15 s; followed by 1 min of annealing/extension at 60 °C. The levels of TSLP mRNA were normalized to those of GAPDH mRNA (the internal control; ΔCt method) and were calculated and displayed as 2^−ΔCt^ values.

### Statistical analysis

Statistical analysis was carried out using the SPSS (version 19.0) software package. The variables were expressed as the mean ± standard deviation (SD). One-way ANOVA accompanied by Bonferonni’s post hoc test for multiple comparisons were utilized to compare differences between groups. Values of P < 0.05 were considered to be statistically significant.

## Additional Information

**How to cite this article**: Dong, H. *et al*. Distinct roles of short and long thymic stromal lymphopoietin isoforms in house dust mite-induced asthmatic airway epithelial barrier disruption. *Sci. Rep.* **6**, 39559; doi: 10.1038/srep39559 (2016).

**Publisher's note:** Springer Nature remains neutral with regard to jurisdictional claims in published maps and institutional affiliations.

## Supplementary Material

Supplementary Information
