# Supplementary material for: Distinct roles of short and long thymic stromal lymphopoietin isoforms in house dust mite-induced asthmatic airway epithelial barrier disruption
Source: Sci Rep. 2016 Dec 20;6:39559. doi: 10.1038/srep39559 (PMC5171874; doi:10.1038/srep39559)
Supplement: Supplementary Information [file srep39559-s1.pdf]

Distinct roles of short and long thymic stromal lymphopoietin isoforms in house dust mite-induced asthmatic airway epithelial barrier disruption.

Hangming Dong[1+];Yahui Hu[1+];Laiyu Liu[1+];Mengchen Zou[1];Chaowen Huang[1]; Lishan Luo[1];Changhui Yu[2];Xuan Wan[1]; Haijin Zhao[1];JiaLong Chen[2];Zhefan Xie[1];Yanqing Le[1];Fei Zou[2**]; Shaoxi Cai[1*]

Figure 1

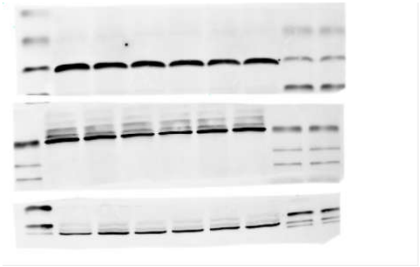

β-actin β-catenin E-cadherin Figure 1 a

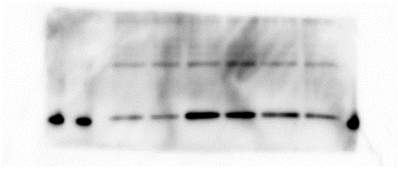

lfTSLP Figure 1 b

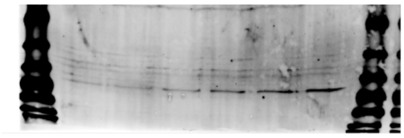

Secreted lfTSLP Figure 1 b

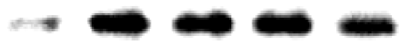

lfTSLP Figure 1 c

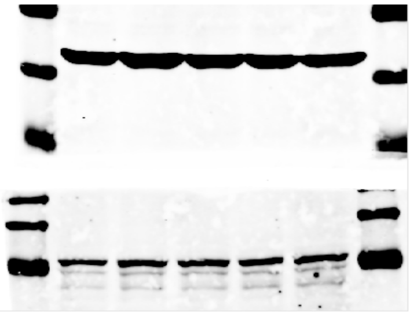

β-actin E-cadherin Figure 1 c

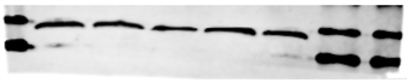

β-catenin Figure 1 c

Figure 2

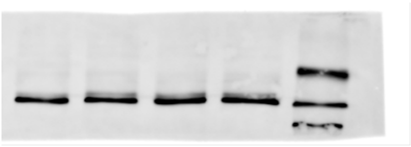

E-cadherin Figure 2 b

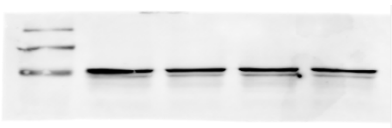

β-catenin Figure 2 b

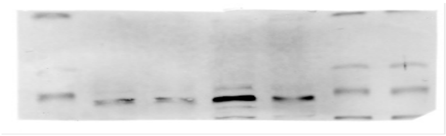

lfTSLP   Figure 2 b

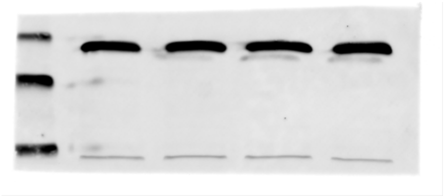

β-actin   Figure 2 b

Figure 3

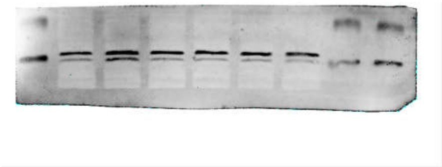

p-ERK   Figure 3 a

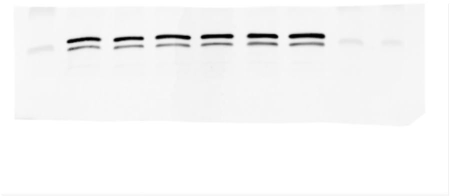

t-ERK   Figure 3 a

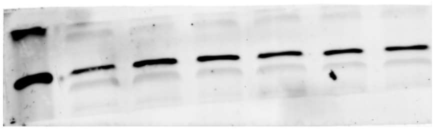

p-p38   Figure 3 a

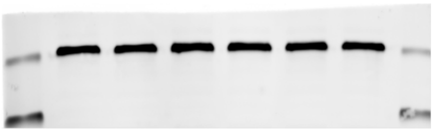

t-p38   Figure 3 a

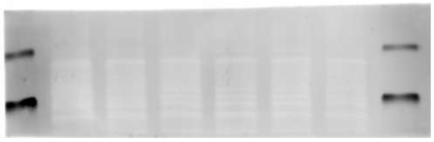

p-JNK   Figure 3 a

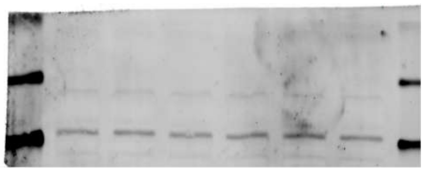

t-JNK   Figure 3 a

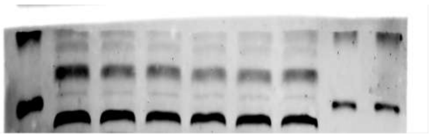

β-actin   Figure 3 a

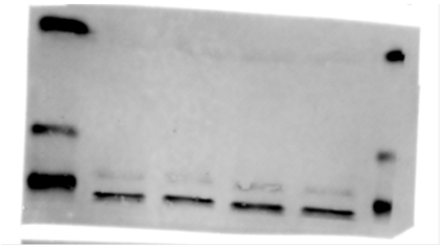
lfTSLP Figure 3 c

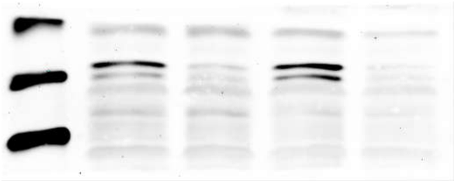
p-ERK Figure 3 c

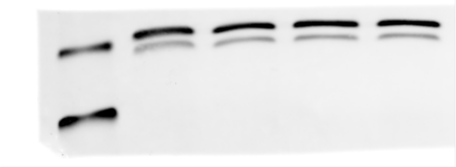
t-ERK Figure 3 c

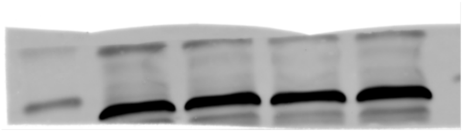
β-actin Figure 3 c

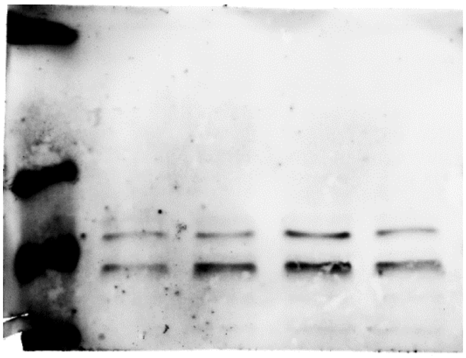
lfTSLP Figure 3 d

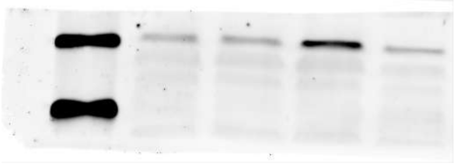
p-p38 Figure 3 d

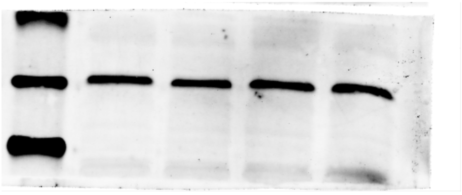
t-p38 Figure 3 d

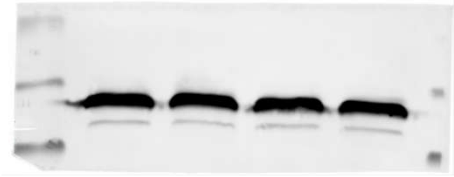
β-actin Figure 3 d

Figure 4

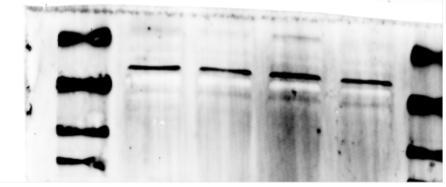

p-STAT5   Figure 4 a

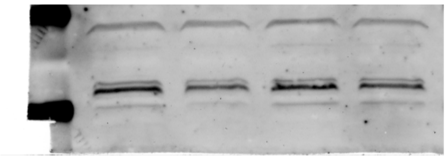

t-STAT5   Figure 4 a

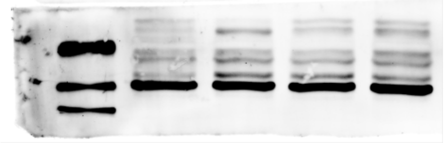

E-cadherin   Figure 4 a

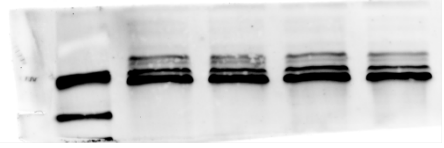

β-catenin   Figure 4 a

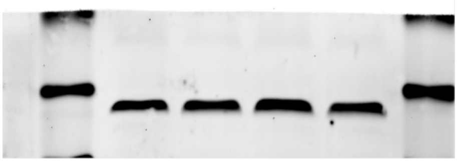

β-actin   Figure 4 a

Figure 5

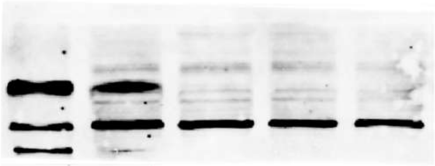

E-cadherin   Figure 5 a

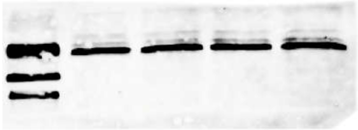

β-catenin   Figure 5 a

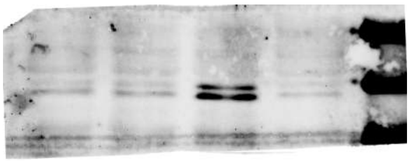

p-ERK   Figure 5 a

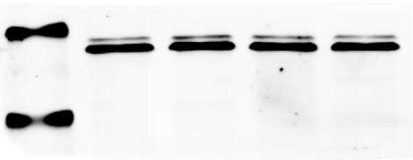

t-ERK   Figure 5 a

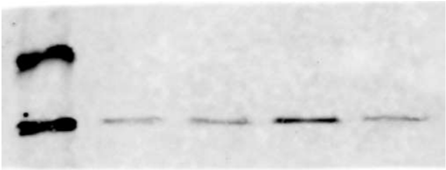

p-p38 Figure 5 a

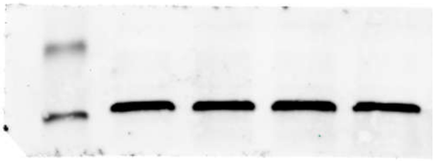

t-p38 Figure 5 a

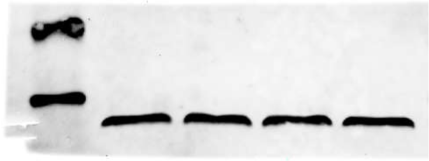

β-actin Figure 5 a

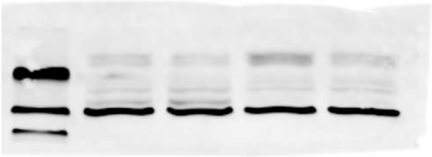

E-cadherin Figure 5 b

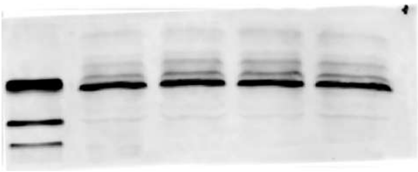

β-catenin Figure 5 b

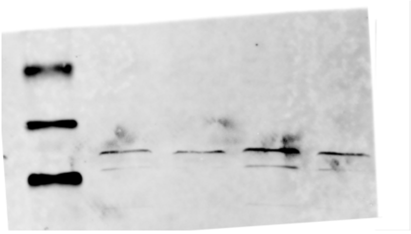

p-STAT5 Figure 5 b

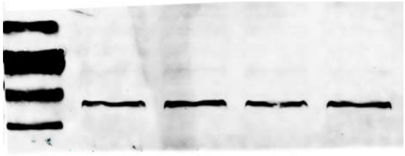

t-STAT5 Figure 5 b

Figure 8

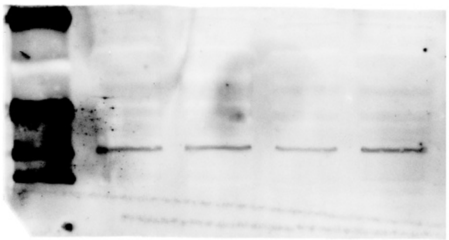

E-cadherin Figure 8 a

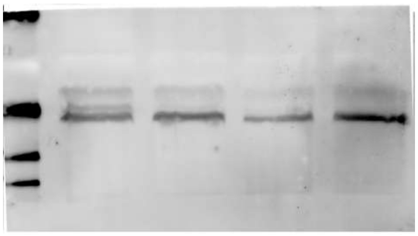

β-catenin Figure 8 a

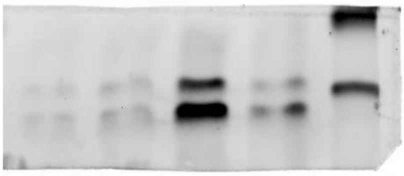

p-ERK Figure 8 a

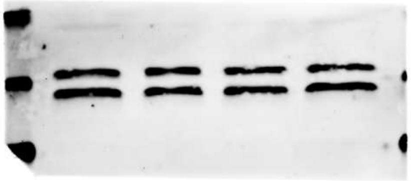

t-ERK Figure 8 a

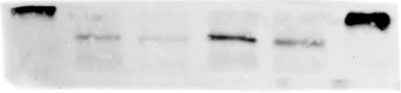

p-p38 Figure 8 a

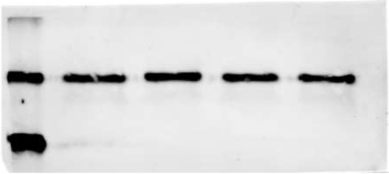

t-p38 Figure 8 a

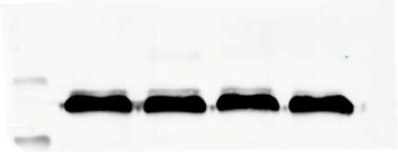

β-actin Figure 8 a

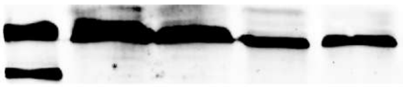

E-cadherin Figure 8 b

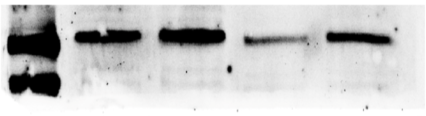

β-catenin Figure 8 b

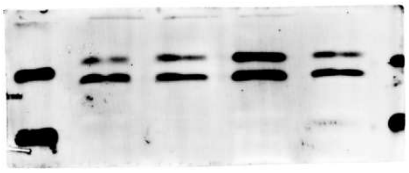

p-ERK Figure 8 b

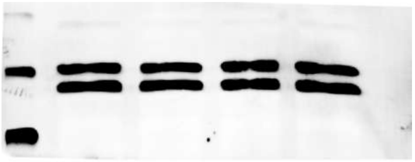

t- ERK Figure 8 b

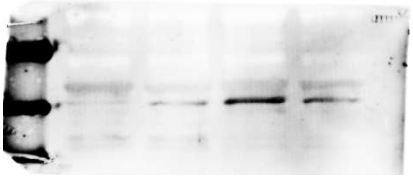

p-p38 Figure 8 b

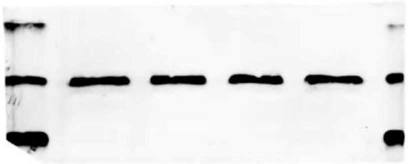

t-p38 Figure 8 b

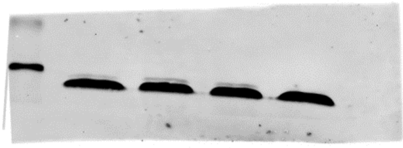

β-actin Figure 8 b
